# Shock index, modified shock index, age shock index score, and reverse shock index multiplied by Glasgow Coma Scale predicting clinical outcomes in traumatic brain injury: Evidence from a 10-year analysis in a single center

**DOI:** 10.3389/fmed.2022.999481

**Published:** 2022-11-22

**Authors:** Po-Chen Lin, Chi-Yuan Liu, I-Shiang Tzeng, Tsung-Han Hsieh, Chun-Yu Chang, Yueh-Tseng Hou, Yu-Long Chen, Da-Sen Chien, Giou-Teng Yiang, Meng-Yu Wu

**Affiliations:** ^1^Department of Emergency Medicine, Taipei Tzu Chi Hospital, Buddhist Tzu Chi Medical Foundation, New Taipei City, Taiwan; ^2^Department of Emergency Medicine, School of Medicine, Tzu Chi University, Hualien City, Taiwan; ^3^Department of Orthopedic Surgery, Taipei Tzu Chi Hospital, Buddhist Tzu Chi Medical Foundation, New Taipei City, Taiwan; ^4^Department of Orthopedics, School of Medicine, Tzu Chi University, Hualien City, Taiwan; ^5^Department of Research, Taipei Tzu Chi Hospital, Buddhist Tzu Chi Medical Foundation, New Taipei City, Taiwan; ^6^Department of Anesthesiology, Taipei Tzu Chi Hospital, Buddhist Tzu Chi Medical Foundation, New Taipei City, Taiwan; ^7^Department of Anesthesiology, School of Medicine, Tzu Chi University, Hualien City, Taiwan

**Keywords:** rSIG, traumatic brain injury, shock index, mortality, prediction

## Abstract

**Objectives:**

Early identification of traumatic brain injury (TBI) patients at a high risk of mortality is very important. This study aimed to compare the predictive accuracy of four scoring systems in TBI, including shock index (SI), modified shock index (MSI), age-adjusted shock index (ASI), and reverse shock index multiplied by the Glasgow Coma Scale (rSIG).

**Patients and methods:**

This is a retrospective analysis of a registry from the Taipei Tzu Chi trauma database. Totally, 1,791 patients with TBI were included. We investigated the accuracy of four major shock indices for TBI mortality. In the subgroup analysis, we also analyzed the effects of age, injury mechanism, underlying diseases, TBI severity, and injury severity.

**Results:**

The predictive accuracy of rSIG was significantly higher than those of SI, MSI, and ASI in all the patients [area under the receiver operating characteristic curve (AUROC), 0.710 vs. 0.495 vs. 0.527 vs. 0.598], especially in the moderate/severe TBI (AUROC, 0.625 vs. 0.450 vs. 0.476 vs. 0.529) and isolated head injury populations (AUROC 0.689 vs. 0.472 vs. 0.504 vs. 0.587). In the subgroup analysis, the prediction accuracy of mortality of rSIG was better in TBI with major trauma [Injury Severity Score (ISS) ≥ 16], motor vehicle collisions, fall injury, and healthy and cardiovascular disease population. rSIG also had a better prediction effect, as compared to SI, MSI, and ASI, both in the non-geriatric (age < 65 years) and geriatric (age ≥ 65 years).

**Conclusion:**

rSIG had a better prediction accuracy for mortality in the overall TBI population than SI, MSI, and ASI. Although rSIG have better accuracy than other indices (ROC values indicate poor to moderate accuracy), the further clinical studies are necessary to validate our results.

## Introduction

Traumatic injury is a major cause of death and disability, causing a major global burden (1). The early identification of patients with trauma at a high risk of mortality is very important for emergency medical technicians and physicians (2). Many studies have investigated and published prognostic predictive models for patients with trauma, including the Injury Severity Score (ISS) (3), Revised Trauma Score (RTS) (4), Trauma and Injury Severity Score (TRISS) (5), and New Injury Severity Score (NISS) (6). In a study of multitrauma patients conducted by Mehmet Hilmi Höke et al. (7), the TRISS had the best performance score in determining mortality, with an area under the receiver operating characteristic curve (AUROC) curve of 0.93, followed by NISS, ISS, and RTS.

Although these scoring systems are effective in predicting survival probability, they can only be calculated using the information of all injured organs, which is usually available at discharge. However, application of TRISS, NISS, ISS, and RTS in prehospital or emergency departments (ED) is limited. In addition, TRISS, NISS, ISS, and RTS involve complicated equations and calculations, which may not reflect real-time patient conditions; moreover, all these scoring systems are difficult to memorize and use in prehospital prediction. A quick and easy tool that provides real-time risk stratification is important in populations with traumatic injury.

The shock index (SI) was developed as a physiological triage score, calculated by the triage heart rate (HR) and systolic blood pressure (SBP) using the following formula: SI = HR/SBP. The SI effectively reflects the hemodynamic status as increased heart rate and decreased systolic blood pressure in the patients with shock, and is associated with higher mortality rates (8). However, the SI may underestimate the severity of underlying shock in different subgroups because only SBP is used for the analysis.

Ye-cheng Liu et al. (9) conducted a retrospective database review of 22,161 patients emergency patients with administration of intravenous fluids, showing modified shock index (MSI) is associated with mortality that is superior to SI. The MSI used mean arterial pressure (MAP) instead of SBP, as the following formula: MSI = HR/MAP. Diastolic blood pressure (DBP) is as important as SBP for determining clinical severity. Terceros-Almanza et al. (10) included 287 severe trauma (ISS > 15) patients and found that the MSI was a good predictor of massive bleeding, with an AUROC of up to 0.90 at an optimal cutoff of 1.46.

In the older population, patients tend to have higher baseline SBPs; therefore, SI multiplied by age (ASI) may be a better predictor than SI. In the study by Zarzaur et al. (11, 12) retrospectively analyzed 189,574 trauma patients from National Trauma Data Bank, showing ASI showed a better prediction effect for 48-h mortality (AUROC, 0.693) in older patients as compared to heart rate, SBP, and SI.

The scores in the novel scoring system, the reverse shock index multiplied by the Glasgow Coma Scale (rSIG) score, were calculated as follows: rSIG = Glasgow Coma Scale (GCS)/SI, which reflects the hemodynamic and neurological conditions that predict mortality in patients with trauma (13). A retrospective, multicenter study analyzed 168,517 patients registered in the Japan Trauma Data Bank, showing the highest AUROC for mortality was 0.901 in the younger population and 0.845 in the older population (13, 14). The rSIG score combines both hemodynamic (shock index) and neurologic status (GCS score) to improve the accuracy of prediction.

Although the prediction accuracies of SI, MSI, ASI, and rSIG for clinical outcomes seem good, few studies have focused on the traumatic brain injury (TBI) population. Trauma patients commonly experience both hypovolemic shock and TBI. A bimodal relationship has been reported between SI and mortality in head-injured but not in non-head injured patients (15). Huai-Kuan Huang et al. (16) also reported the bimodal relationship between blood pressure and mortality from total of 1782 traumatic adult patients with TBI (AIS score < 3). The SI altered the predictability of TBI owing to autonomic uncoupling and significantly underestimated underlying hemorrhage in the acute TBI population (17).

In this study, we aimed to analyze the prediction accuracies of four modified SI scoring systems in TBI, including SI, MSI, ASI, and rSIG. We conducted a retrospective cohort study of TBI injury from a registry of Taipei Tzu Chi trauma database and we also hypothesized that the rSIG would have a better prediction performance for mortality in the TBI population.

## Materials and methods

### Study design and setting

This retrospective cohort study was approved by the Institutional Review Board of Taipei Tzu Chi Hospital (IRB number: 10-XD-070), and patient data were retrospectively reviewed from the Taipei Tzu Chi Hospital trauma database, which included patients with traumatic injury (coding ICD-9-CM codes 800–959, excluding 905–909 and 930–939, or coding ICD-10-CM codes S00–T98, excluding T15–T19 and T90–T98) with hospitalization and major trauma. A total of 152 data variables were analyzed, including detailed patient demographics, prehospital medical conditions, prehospital vital signs, in-hospital triage vital signs, abbreviated injury scale (AIS) score, ISS, admission condition, and in-hospital mortality.

### Selection of participants

We included patients with TBI with head AIS score ≥3 from the Taipei Tzu Chi Hospital trauma database between January 2009 and June 2019. The exclusion criterion was age <20 years. Many trauma scoring systems were investigated and their clinical outcomes were predicted, including SI, MSI, ASI, rSIG, ISS, GCS, RTS, SI, NISS, and TRISS (based on the ISS, RTS, age, and injury mechanism). The patients were separated into two groups, survical and mortality groups, for analysis. A subgroup analysis was conducted to analyze the traumatic score systems in different age groups, populations, injury types, and severities of TBI.

### Variable measurements

The basic characteristics of the trauma injury population in our study included age, sex, underlying diseases, triage level, in-hospital triage vital signs, GCS score, mechanism of injury, injury site, and pre-hospital management (neck collar on arrival, fixing with spinal board, and wearing a helmet). In-hospital triage vital signs including heart rate, SBP, DBP, and respiratory rate were recorded when patients arrived at the hospital and were used to calculate the scores in the four scoring systems.

In the subgroup analysis, we adopted ISS as the index of trauma severity and defined patients with ISS scores ≥ 16 as the major trauma population. TBI was divided into mild TBI (GCS score > 13), moderate/severe TBI (GCS score ≤ 13), and isolated TBI (head AIS score ≥ 3 and any AIS score = 0). The patients were also divided into non-geriatric (age < 65 years) and geriatric (age ≥ 65 years) groups for the subgroup analysis.

### Outcomes

The primary outcome was in-hospital mortality rate. In case of in-hospital death, the time of death was recorded. The secondary outcomes included intensive care unit (ICU) admission, ICU readmission, length of stay (LOS) in the ICU, prolonged ICU stay (defined as ICU admission duration > 14 days), total hospital LOS, need for surgery, and reoperation.

### Statistical analysis

Detailed demographic, overall survival, and clinical outcome data were statistically analyzed using SPSS software (Version 13.0 SPSS Inc, Chicago, IL, USA). The numbers of cases with missing values for diastolic blood pressure (1 cases), respiratory rate (7 cases), MSI (1 cases), RTS (7 cases, due to missing value of RR), TRISS (7 cases, due to missing value of RR) were trivial (totally 8 cases, 0.45%), so imputation was not considered necessary (18). All continuous data were subjected to the Kolmogorov–Smirnov test for testing the normal distribution. All the continuous variables are reported as mean ± standard deviation (SD), and non-normally distributed data are presented as medians with interquartile ranges (IQR). Categorical variables are presented as numbers and percentages. Independent sample t-test or Mann–Whitney U test was used for continuous variables. Categorical and nominal variables were compared using Pearson chi-squared test or Fisher’s exact test. One-way analysis of variance was used for analysis of four major shock index score in mild, moderate, and severe traumatic brain injury groups. Univariable and multivariable logistic regression were used to determine the potential factors that associated to primary and secondary outcomes in patients with trauma. Variables with *p*-values < 0.2 or important variables were selected for multivariable logistic regression analysis. The AUROC was used for each outcome to analyze the discrimination of the regression model. The performance of scoring systems for mortality prediction, including sensitivity, specificity, positive predictive value, negative predictive value, and accuracy, were calculated. The best cut-of values of scoring systems were identified by Youden index. All the tests were 2-sided, and *p* values < 0.05 were considered statistically significant.

## Results

### Characteristics of study objects

**FIGURE 1:**
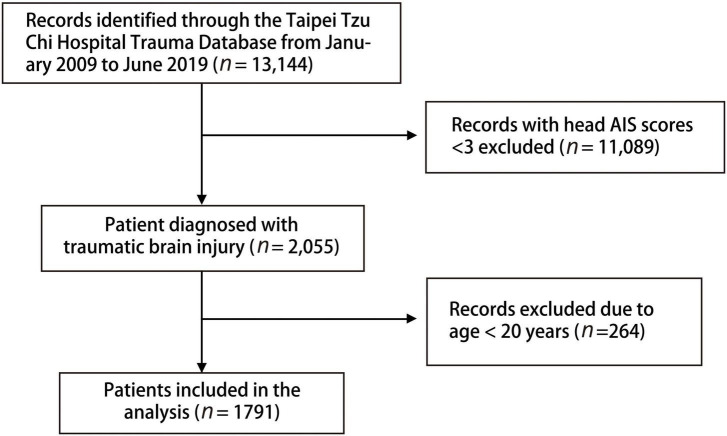
Flow diagram of patients included in our study.

A total of 13,144 patients in the database were eligible for the analysis, and 11,089 who did not have head AIS score ≥ 3 or who were aged < 20 years (*n* = 264) were excluded. Finally, 1,791 patients with brain injury [aged (mean ± SD), 63.3 ± 19.8 years; 60.5% men] were included in this study (Figure 1). The in-hospital mortality rate in this study was 9.3% (Table 1). Cardiovascular disease was the main underlying disease in the survival and mortality groups. In the mortality group, the triage level was higher, with 58.4% triage level I and 34.9% triage level II. The mortality group were more likely to be placed in a neck collar and fixed to a spinal board. Traffic accidents and falls were common injury mechanisms in our database, with motorcycle accidents accounting for 39.9%. Helmets were worn by 66.5% of all patients. In the clinical outcome analysis, the mortality group had a higher proportion of ICU admissions, readmissions, and prolonged ICU hospitalizations. However, longer total LOS and higher operation rates were noted in the survival group.

**TABLE 1:** Demographics of the traumatic brain injury population.

Characteristics	Total patients	Survival	Death	*p*-value
Patient number, *n* (%)	1,791 (100.0%)	1,625 (90.7%)	166 (9.3%)	
Age (years), mean ± SD	63.33 ± 19.82	62.77 ± 19.72	68.83 ± 19.99	< 0.001
Gender, *n* (%)				0.349
Male	1083 (60.5%)	977 (60.1%)	106 (63.9%)	
Female	708 (39.5%)	648 (39.9%)	60 (36.1%)	
Underlying diseases, *n* (%)				
CNS diseases	169 (9.4%)	153 (9.4%)	16 (9.6%)	0.925
Cardiovascular diseases	613 (34.2%)	554 (34.1%)	59 (35.5%)	0.708
Respiratory diseases	36 (2.0%)	31 (1.9%)	5 (13.0%)	0.334
Chronic kidney disease	65 (3.6%)	54 (3.3%)	11 (6.6%)	0.030
Diabetes mellitus	273 (15.2%)	250 (15.4%)	23 (13.9%)	0.602
Triage level, *n* (%)				< 0.001
I	413 (23.1%)	316 (19.4%)	97 (58.4%)	
II	996 (55.6%)	938 (57.7%)	58 (34.9%)	
III	376 (21.0%)	365 (22.5%)	11 (26.6%)	
IV and V	6 (0.3%)	6 (0.4%)	0 (0.0%)	
Triage vital sign				
SBP (mmHg, mean ± SD)	154.93 ± 35.23	154.23 ± 34.36	161.72 ± 42.40	0.029
*DBP (mmHg, mean ± SD)	87.46 ± 18.00	87.53 ± 17.50	86.79 ± 22.36	0.678
*RR (per minutes, mean ± SD)	18.62 ± 2.76	18.72 ± 2.42	17.67 ± 4.95	0.008
HR (per minutes, mean ± SD)	86.47 ± 18.25	85.88 ± 17.51	92.25 ± 23.64	0.001
GCS, median (IQR)	15 (13–15)	15 (14–15)	8 (3.75–15)	< 0.001
Injury site, *n* (%)				
Home	643 (35.9%)	570 (35.1%)	73 (44.0%)	0.023
Street	917 (51.2%)	841 (51.8%)	76 (45.8%)	0.143
Public site	158 (8.8%)	146 (9.0%)	12 (7.2%)	0.447
Others	73 (4.1%)	68 (4.2%)	5 (3.0%)	0.467
Mechanism of injury, *n* (%)^‡^				
MVC	715 (39.9%)	647 (39.8%)	68 (41.0%)	0.774
Falling	997 (55.7%)	902 (55.5%)	95 (57.2%)	0.671
Traumatic brain injury (TBI), *n* (%)				
Mild TBI	1375 (76.8%)	1319 (81.2%)	56 (33.7%)	< 0.001
Moderate TBI	182 (10.2%)	157 (9.7%)	25 (15.1%)	0.028
Severe TBI	234 (3.1%)	149 (9.2%)	85 (51.2%)	< 0.001
Isolated TBI	1378 (76.9%)	1254 (77.2%)	124 (74.7%)	0.472
Neck collar on arrival *n* (%)	477 (26.6%)	403 (24.8%)	74 (44.6%)	< 0.001
Fixed with spinal board, *n* (%)	317 (17.7%)	261 (16.1%)	56 (33.7%)	< 0.001
Had wear a helmet, *n* (%)	453 (66.5%)	420 (67.2%)	33 (58.9%)	0.209
Scoring systems				
Shock index, mean ± SD	0.59 ± 0.21	0.59 ± 0.20	0.63 ± 0.30	0.089
Shock index > 0.9, n (%)	121 (6.8%)	96 (5.9%)	25 (15.1%)	< 0.001
MSI, mean ± SD	0.82 ± 0.27	0.82 ± 0.26	0.89 ± 0.38	0.001
Age SI, mean ± SD	36.15 ± 14.85	35.69 ± 14.61	40.60 ± 16.39	< 0.001
rSIG, mean ± SD	24.72 ± 10.08	25.52 ± 9.57	16.96 ± 11.59	< 0.001
ISS, median (IQR)	11 (9–16)	10 (9–16)	18 (10–25)	< 0.001
ISS ≥ 16, *n* (%)	728 (40.6%)	605 (37.2%)	123 (74.1%)	< 0.001
RTS, mean ± SD	7.30 ± 1.24	7.45 ± 1.02	5.85 ± 2.01	< 0.001
NISS, mean ± SD	15.73 ± 9.63	14.81 ± 8.25	24.68 ± 15.71	< 0.001
TRISS, mean ± SD	0.90 ± 0.20	0.92 ± 0.16	0.67 ± 0.33	< 0.001
Clinical outcome				
LOS days, median (IQR)	9 (5–19)	9 (5–19)	5 (3–12.25)	< 0.001
ICU Admission, *n* (%)	1238 (69.1%)	1091 (67.1%)	147 (88.6%)	< 0.001
ICU Readmission, n(%)	33 (1.8%)	28 (1.7%)	5 (3.0%)	0.239
ICU days, median (IQR)	3 (0–6)	3 (0–5)	5 (2–9)	< 0.001
ICU LOS ≥ 14 days, *n* (%)	79 (4.4%)	69 (4.2%)	10 (6.0%)	0.288
Operation, *n* (%)	443 (24.7%)	412 (25.4%)	31 (18.7%)	0.057
Reoperation, *n* (%)	72 (4.0%)	71 (4.4%)	1 (0.6%)	0.012

CNS diseases, central nervous system disease; SBP, systolic blood pressure; DBP, diastolic blood pressure; RR, respiration rate; HR, heart rate; GCS, Glasgow Coma Scale; MCV, motor vehicle collision; MSI, modified shock index; ASI, age-adjusted shock index; rSIG, reverse shock index multiplied by Glasgow Coma Scale; ISS, Injury Severity Score; RTS, Revised Trauma Score; NISS, New Injury Severity Score; TRISS, Trauma and Injury Severity Score; LOS, length of stay; ICU, intensive care unit. *The total missing cases was 8, comprising a single value of DBP for 1 patient that survived, and RR for 7 patients (6 in the survival group and 1 in the mortality group). ^‡^Mechanism of injury: there was one patient with gunshot injury. No patients with blast injury was included.

### Injury Severity Score, Revised Trauma Score, New Injury Severity Score, and Trauma and Injury Severity Score

**FIGURE 2:**
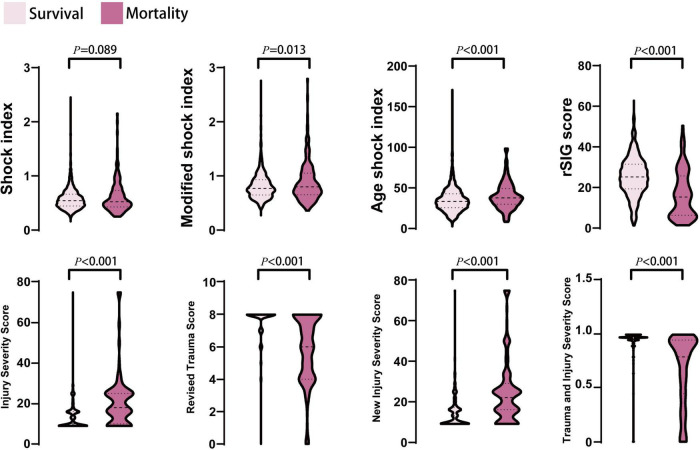
The distribution and density of all prediction score systems in survival and mortality groups was illustrated in violin plot. Inside the violin plot interquartile ranges and outlier whiskers are presented as a thick and thin line equivalent to a boxplot. There were no statistical differences for shock index between survival and mortality groups.

In the mortality group, the ISS and NISS were significantly higher, and the RTS and probability of survival from TRISS were significantly lower than those in the survival group (Table 1 and Figure 2). The median ISS (ISS ≥ 16) was significantly higher in the mortality group (up to 74.1% of patients). In logistic regression analysis, the ISS, RTS, NISS, and TRISS were significant predictors of mortality, ICU admission and prolonged ICU stay (Table 2). The TRISS was the best predictor of all scoring systems in all subgroup populations. The predictive accuracy of ISS and NISS were better in moderate/severe TBI than mild TBI population. RTS was not a significant predictor of mortality in mild TBI group. The prediction performance of ISS, RTS and NISS were also better in major injury (ISS ≥ 16) than mild injury (ISS < 16) population. ISS, RTS, NISS and TRISS are effective for prediction of mortality in both younger and older population.

**TABLE 2:** Clinical outcome prediction by univariable logistic regression.

Characteristics	Odds ratio of death			Odds ratio of ICU admission			Odds ratio of prolonged stay		
	OR	95% CI	*p*–Value	OR	95% CI	*p*–Value	OR	95% CI	*p*–Value
Age	1.017	1.008–1.026	< 0.001	1.003	0.998–1.008	0.236	1.013	1.001–1.026	0.036
Female	0.853	0.612–1.189	0.349	1.515	1.236–1.857	< 0.001	1.013	0.638–1.607	0.957
Underlying diseases									
Cardiovascular diseases	1.066	0.763–1.489	0.708	0.947	0.767–1.169	0.610	1.254	0.790–1.991	0.338
Chronic kidney disease	2.065	1.058–4.031	0.034	1.510	0.840–2.712	0.168	0.330	0.045–2.411	0.275
Diabetes mellitus	0.885	0.558–1.402	0.602	1.027	0.776–1.358	0.854	1.208	0.668–2.185	0.531
Activation of trauma team	4.418	3.026–6.450	< 0.001	0.230	0.141–0.374	< 0.001	0.273	0.162–0.458	< 0.001
Triage level									
I	Ref	Ref	Ref	Ref	Ref	Ref	Ref	Ref	Ref
II	0.201	0.142–0.286	< 0.001	3.630	0.648–20.331	0.142	–	–	–
III	0.098	0.052–0.186	< 0.001	1.018	0.186–5.588	0.983	–	–	–
Vital sign									
SBP	1.006	1.001–1.010	0.009	1.004	1.001–1.007	0.008	1.003	0.997–1.010	0.286
DBP	0.998	0.989–1.007	0.612	1.002	0.996–1.007	0.529	0.984	0.971–0.997	0.014
RR	0.897	0.856–0.941	< 0.001	1.011	0.975–1.048	0.562	1.047	0.961–1.142	0.294
HR	1.017	1.009–1.025	< 0.001	1.010	1.004–1.016	0.001	1.012	1.001–1.024	0.034
GCS	0.764	0.736–0.793	< 0.001	0.798	0.760–0.838	< 0.001	0.861	0.820–0.904	< 0.001
Injury site									
Home	1.453	1.052–2.007	0.023	0.803	0.653–0.988	0.038	1.524	0.968–2.399	0.069
Street	0.787	0.571–1.085	0.143	1.147	0.939–1.402	0.179	0.749	0.475–1.179	0.211
Public site	0.789	0.428–1.455	0.448	1.215	0.843–1.751	0.297	0.844	0.361–1.971	0.694
Mechanism of injury, n (%)									
MVC	1.049	0.758–1.451	0.774	1.271	1.033–1.564	0.023	0.867	0.543–1.385	0.551
Falls	1.072	0.777–1.481	0.671	0.833	0.680–1.020	0.077	1.178	0.744–1.865	0.484
Neck collar on arrival, n (%)	2.439	1.760–3.379	< 0.001	2.016	1.572–2.585	< 0.001	1.833	1.152–2.917	0.011
Full spinal immobilization, n (%)	2.661	1.879–3.768	< 0.001	2.258	1.665–3.062	< 0.001	2.251	1.378–3.677	0.001
Wearing a helmet, *n* (%)	0.700	0.401–1.224	0.211	0.657	0.455–0.946	0.024	1.138	0.487–2.658	0.765
Scoring systems									
Shock index	2.140	1.128–4.061	0.020	1.149	0.715–1.847	0.566	1.655	0.653–4.197	0.288
Shock index > 0.9	2.824	1.760–4.530	< 0.001	0.793	0.523–1.203	0.276	2.090	1.048–4.170	0.036
MSI	2.308	1.415–3.765	0.001	1.296	0.882–1.904	0.187	2.180	1.124–4.231	0.021
ASI	1.019	1.009–1.028	< 0.001	1.005	0.998–1.012	0.149	1.010	0.996–1.023	0.160
rSIG	0.911	0.895–0.928	< 0.001	0.968	0.959–0.978	< 0.001	0.956	0.934–0.978	< 0.001
ISS	1.097	1.075–1.118	< 0.001	1.077	1.055–1.098	< 0.001	1.045	1.026–1.065	< 0.001
ISS ≥ 16	4.832	3.369–6.923	< 0.001	2.922	2.334–3.658	< 0.001	2.785	1.739–4.460	< 0.001
RTS	0.540	0.487–0.598	< 0.001	0.658	0.577–0.749	< 0.001	0.735	0.656–0.823	< 0.001
NISS	1.069	1.055–1.083	< 0.001	1.068	1.051–1.086	< 0.001	1.040	1.024–1.055	< 0.001
Probability of survival^‡^	0.031	0.018–0.054	< 0.001	0.193	0.095–0.389	< 0.001	0.295	0.130–0.672	0.004

SBP, systolic blood pressure; DBP, diastolic blood pressure; RR, respiration rate; HR, heart rate; GCS, Glasgow Coma Scale; MCV, motor vehicle collision; MSI, modified shock index; ASI, age-adjusted shock index; rSIG, reverse shock index multiplied by Glasgow Coma Scale; ISS, Injury Severity Score; RTS, Revised Trauma Score; NISS, New Injury Severity Score; TRISS, Trauma and Injury Severity Score. ^‡^Probability of survival: which is the output of the TRISS process based on ISS, RTS, age, and injury mechanism.

### Shock index, modified shock index, age-adjusted shock index, and reverse shock index multiplied by Glasgow Coma Scale score

**FIGURE 3:**
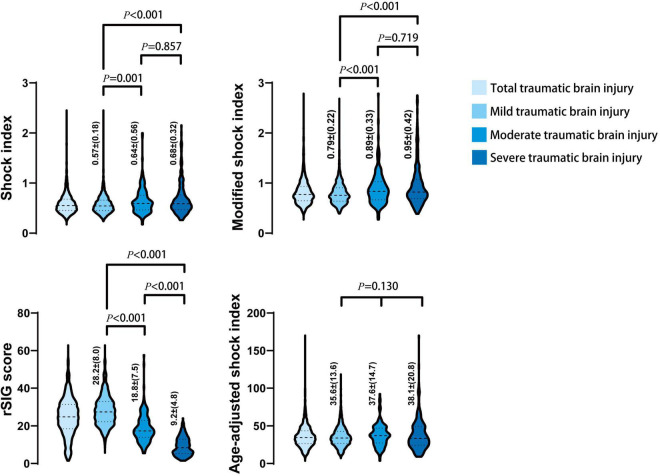
The distribution and density of four prediction score systems in different severity of traumatic brain injury groups was illustrated in violin plot. Inside the violin plot interquartile ranges and outlier whiskers are presented as a thick and thin line equivalent to a boxplot. The detail score of shock index, modified shock index, rSIG, and age-adjusted shock index, in mild, moderate, and severe TBI was showed beside each violin as mean ± (standard deviation).

In the mortality group, the MSI, and ASI scores were significantly higher, and the rSIG score was significantly lower than those in the survival group (Table 1 and Figure 2). In Figure 3, the subgroup analysis of brain injury severity showed that the SI, MSI, and ASI scores were higher in the severe TBI group (SI, 0.68 ± 0.32; MSI, 0.95 ± 0.42; and, ASI, 38.1 ± 20.8), followed by the moderate (SI, 0.64 ± 0.56; MSI, 0.89 ± 0.33; and, ASI, 37.6 ± 14.7) and mild TBI groups (SI, 0.57 ± 0.18; MSI, 0.79 ± 0.22; and, ASI, 35.6 ± 13.6). In the rSIG analysis, the patients with severe TBI presented with significantly lower rSIG scores than the patients with mild and moderate TBI. There were no significant differences in the SI, MSI, and ASI scores between the moderate- and severe-TBI populations.

### Predictive accuracy for clinical outcomes

The results showed that ISS, RTS, NISS, TRISS, SI, MSI, ASI, and rSIG were significant predictors of mortality. In the prolonged ICU analysis, ISS, RTS, NISS, TRISS, MSI and rSIG were effective for prediction. ISS, RTS, NISS, TRISS, and rSIG were significantly associated for the prediction of ICU admission (Table 2). In multivariable logistic regression analysis, ISS, RTS, NISS, TRISS, ASI, and rSIG were significant predictors of mortality and ICU admission. Only rSIG, NISS and TRISS were significantly associated for the prediction of prolonged ICU stay (Table 3).

**TABLE 3:** Clinical outcome prediction by multivariable logistic regression.

Characteristics	Adjusted odds ratio of death			Adjusted odds ratio of ICU admission			Adjusted odds ratio of prolonged stay		
	OR	95% CI	*p*–Value	OR	95% CI	*p*–Value	OR	95% CI	*p*–Value
Scoring systems									
SI	1.487	0.714–3.098	0.289	0.799	0.448–1.426	0.448	1.292	0.499–3.348	0.597
MSI	1.512	0.852–2.684	0.158	0.925	0.584–1.465	0.740	1.714	0.843–3.488	0.137
ASI	1.013	1.003–1.024	0.010	1.007	0.999–1.015	0.084	1.004	0.991–1.018	0.548
rSIG	0.928	0.908–0.949	< 0.001	0.981	0.968–0.993	0.002	0.972	0.945–0.999	0.040
Injury scoring systems									
ISS	1.064	1.040–1.089	< 0.001	1.036	1.014–1.060	0.002	1.021	0.996–1.047	0.100
RTS	0.808	0.677–0.964	0.018	1.339	1.107–1.619	0.003	0.950	0.736–1.225	0.692
NISS	1.043	1.027–1.060	< 0.001	1.037	1.019–1.056	< 0.001	1.020	1.000–1.040	0.051
Probability of survival ^‡^	0.248	0.111–0.556	0.001	2.536	1.154–5.571	0.021	5.785	1.177–28.438	0.031

Co-variables used in the multivariable logistic regression included age, sex, activation of trauma team, injury mechanism, injury type, Glasgow Coma Scale, triage, and prehospital management. Every scoring system was put in the multivariable logistic regression separately from each other due to their strong collinearity. The age was not adjusted in ASI analysis, and GCS was also not adjusted in rSIG analysis due to strong collinearity. ^‡^Probability of survival: which is the output of the TRISS process based on ISS, RTS, age, and injury mechanism.

**FIGURE 4:**
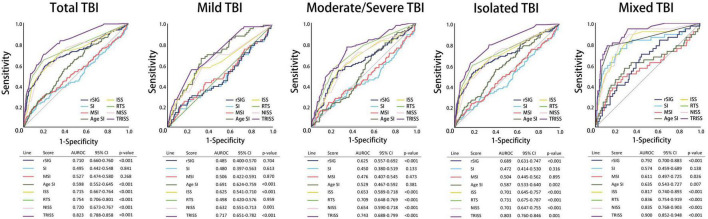
Schematic diagram illustrating detailed AUROC analysis in the total TBI, mild TBI, moderate/severe TBI, isolated TBI and mixed TBI populations.

**FIGURE 5:**
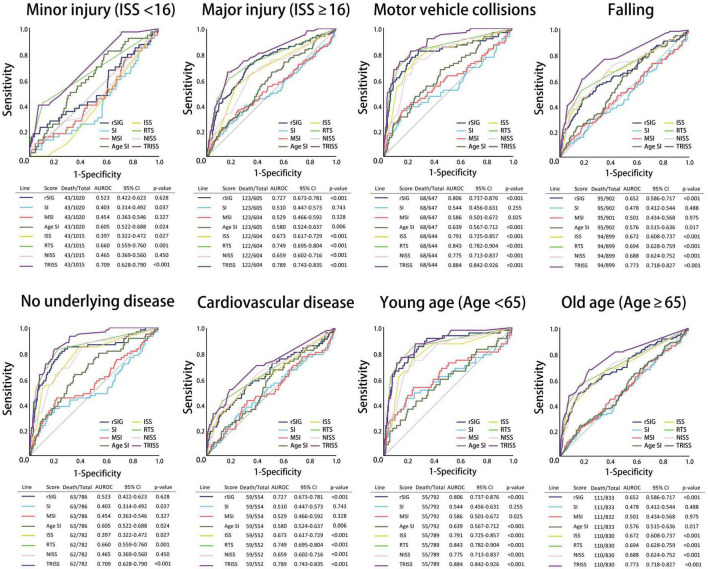
Schematic diagram illustrating detailed AUROC in the subgroup analysis.

All the AUROC for mortality prediction are shown in Figures 4, 5. The predictive accuracy of TRISS was significantly highest in all scoring systems in patients with TBI (AUC: 0.823), mild TBI (AUC: 0.717), moderate/severe TBI (AUC: 0.743), and isolated TBI (AUC: 0.803). ISS, RTS, NISS, and TRISS were significantly effective to predict mortality in patients with TBI, moderate/severe TBI, and isolated TBI. In mild TBI population, RTS was no significantly predicted for mortality with AUC of 0.498 under 95% CI of 0.420–0.576.

The predictive accuracy of rSIG was significantly higher than those of SI, MSI, and ASI in all the patients with TBI (AUC 0.710 vs. 0.495 vs.0.527 vs. 0.598) and in patients with isolated head injury (AUC 0.689 vs. 0.472 vs.0.504 vs. 0.587). We found that ASI was a better predictor of mortality in patients with mild TBI than SI, MSI, and rSIG (AUC 0.691 vs. 0.480 vs. 0.506 vs. 0.485). However, in moderate/severe TBI, rSIG was better than SI, MSI, and ASI (AUC 0.625vs. 0.450 vs.0.476 vs. 0.529).

**FIGURE 6:**
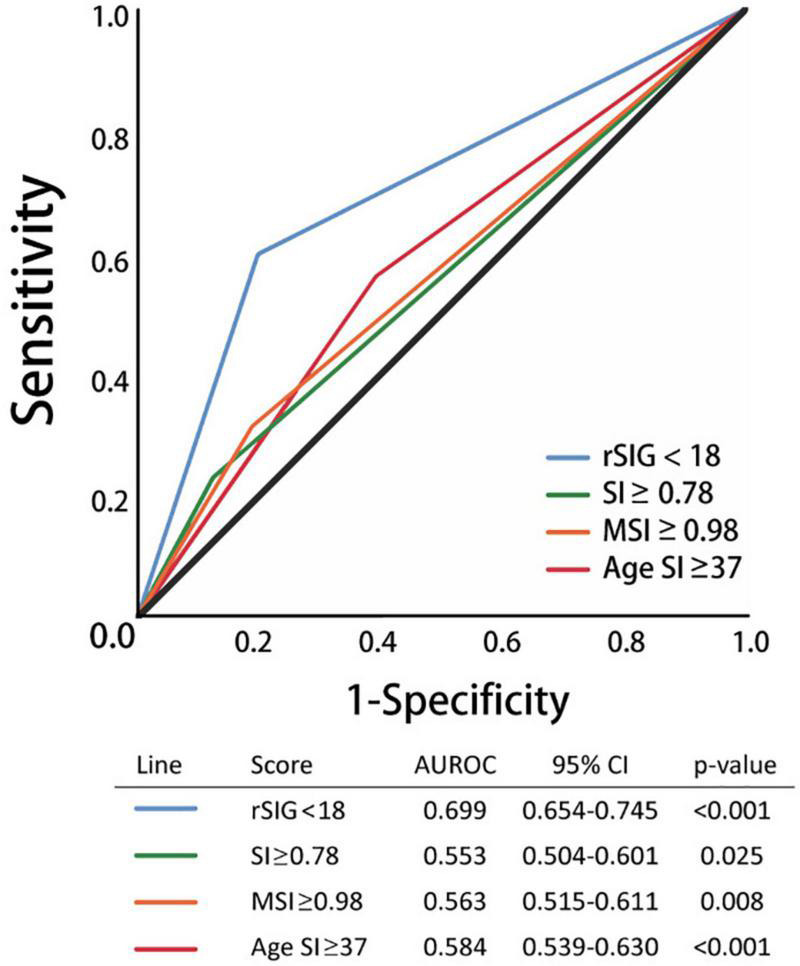
Area under the curve of rSIG < 18, SI ≥ 0.78, MSI ≥ 0.98, and ASI ≥ 37 for mortality prediction.

Our study found that the best rSIG cutoff value was 18 in our study population. The cutoff values of SI, MSI and ASI for mortality were 0.78, 0.98, and 37 (Table 4). The AUROC of rSIG < 18, SI ≥ 0.78, MSI ≥ 0.98, and ASI ≥ 37 for mortality prediction were 0.699, 0.553, 0.563, and 0.584 (Figure 6).

**TABLE 4:** Performance of scoring systems in prediction of mortality.

Scoring system	Cut-off	Sensitivity (95% CI)	Specificity (95% CI)	PPV (95% CI)	NPV (95% CI)	Accuracy (95% CI)
rSIG	18	59.64% (51.76–67.17%)	80.25% (78.23–82.16%)	23.57% (20.83–26.55%)	95.11% (94.17–95.91%)	78.34% (76.35–80.22%)
ASI	37	56.02% (48.12–63.71%)	60.80% (58.38–63.18%)	12.74% (11.19–14.47%)	93.12% (91.90–94.17%)	60.36% (58.05–62.63%)
MSI	0.98	31.33% (24.36–38.97%)	81.22% (79.23–83.09%)	14.57% (11.75–17.91%)	92.04% (91.24–92.78%)	76.59% (74.56–78.54%)
SI	0.78	22.89% (16.74–30.04%)	87.63% (85.93–89.19%)	15.90% (12.20–20.46%)	91.75% (91.09–92.37%)	81.63% (79.76–83.40%)

In the subgroup analysis, we found that the predictive accuracy of TRISS was also significantly highest in all scoring systems in all subgroup populations. ISS, RTS, NISS, and TRISS were significantly effective to predict mortality in major trauma, motor vehicle collisions, falls, no underlying disease, cardiovascular disease, young age, and old age population. NISS was not significantly predicted for mortality in mild injury population with AUC: 0.465 under 95% CI of 0.369–0.560.

In SI, MSI, ASI, and rSIG scoring systems, rSIG had better prediction accuracy of mortality in TBI in the major trauma, motor vehicle collisions, falls, and no underlying disease population. rSIG also had a better prediction effect, as compared to SI, MSI, and ASI, in the older population than that in the younger population.

## Discussion

Our study provided strong evidence that rSIG has a better predictive ability for mortality in the TBI population as compared to SI, MSI, and ASI (AUC 0.710 vs. 0.495 vs. 0.527 vs. 0.598). We also demonstrated that the predictive accuracy of the rSIG was significantly higher in patients with moderate/severe TBI and in patients with isolated head injuries. However, in patients with mild TBI, no difference was found between in the predictive accuracies of the rSIG and the other three scores. This result is consistent with the pathophysiology of TBI. TBI that induces neuronal cell death and cerebral edema may cause increased intracranial pressure (IICP) (19). Severe IICP is associated with hypertension and bradycardia, known as the Cushing triad, which impairs SI accuracy (20, 21). Odom et al. (15) reported a bimodal relationship between SBP and mortality in a TBI population. This result was also found between SI and mortality in patients with head injury, but not in those without head injury.

The rSIG score combines the SI and GCS scores, thereby reflecting hemorrhagic severity and neurologic injury, which may present a synergistic effect with a better prediction rate in TBI. In the moderate/severe-TBI population (GCS < 13), rSIG presented advantages of both the prediction scores, GCS and SI, for mortality. Only in mild TBI, the prediction performance of SI was observed (AUROC rSIG vs. SI: 0.485 vs. 0.480). Patients with moderate/severe TBI would present with the Cushing triad, which is different from hemorrhagic shock presentation. Under these conditions, the prediction accuracy of rSIG was higher than that of SI. In mild TBI, the rSIG and SI may present similar results for the prediction of mortality. In addition, the prediction accuracy of mortality was better in rSIG than those in SI, MSI, and ASI in the major-injury population (ISS ≥ 16), but not in the minor-injury population (ISS < 16). In motor vehicle collisions and fall injuries, rSIG had a better prediction accuracy for mortality than SI, MSI, and ASI.

Although many modified scoring systems have been suggested for improving the prediction accuracy in patients with trauma, few studies have focused on prediction accuracy and application in patients with trauma and TBI. Among these models, TRISS was the best, whereas SI was the worst model for predicting mortality in patients with TBI. The TRISS comprises physiological variables (RTS) and additional information such as age, anatomical variables (ISS), and mechanism (blunt or penetrating) to predict the mortality outcomes. Although it is not surprising that TRISS had better predictive accuracy, it is cumbersome and impractical. rSIG is easier to calculate in emergency departments and during prehospital care. The use of rSIG is as a risk stratification screening tool for high-risk patients instead of substitute TRISS in the prediction of mortality in TBI patients. In our results, we had demonstrated that the addition of consciousness condition would improve SI predictive accuracy and become more suitable in TBI population.

Age is another factor that affects the prediction of mortality. The older population has a less sympathetic-responsive HR and a higher SBP range, which leads to an increase in the false-negative values of SBP as age increases (12, 22). The association between SI ≥ 1 and 30-day mortality risk was weakened in the older population (23). Although the rSIG did not include age for risk assessment, the performance of rSIG was still better than that of AIS in the older population in our subgroup analysis (AUROC of rSIG vs. ASI: 0.694 vs.0.524).

A retrospective multicenter study with 168,517 patients from the Japan Trauma Data Bank by Kimura et al. showed that the predictive accuracy of rSIG for mortality was higher in younger patients (aged < 55 years) than that in older patients (aged ≥ 55 years) with AUROC (95% CI): 0.901 (0.894–0.908) vs. 0.834 (0.828–0.839) (13). In our subgroup analysis, the performance of rSIG showed similar results, with higher predictive accuracy in the younger population (aged < 65 years) for mortality (AUROC up to 0.859), which was almost similar to that of TRISS (AUROC: 0.877, *p* < 0.001). In older patients (aged ≥ 65 years), rSIG also had better predictive accuracy for mortality (AUROC, 0.694) compared to SI, MSI, and ASI (AUROC: 0.502 vs. 0.519 vs. 0.524). These findings also support a study by Lammers et al. (24) of 22,218 adult trauma patients (18 to 65-years-old) from Department of Defense Trauma Registry that showed rSIG had an AUROC as high as that of TRISS in the young population (rSIG AUROC: 0.923 vs. TRISS AUROC: 0.955).

Few studies have reported the effect of underlying diseases in patients with TBI. Our patients with TBI had a high proportion of cardiovascular disease population (up to 34.2%). In these populations, β-blockers and calcium channel blockers were widely used as anti-hypertensives and for decreasing the heart rate, which masked the shock signs required for calculating the SI index. In our subgroup analysis, the AUROC of rSIG in the healthy population after TBI was up to 0.815 (95% CI, 0.750–0.880; *p*-value, < 0.001). In the cardiovascular disease group, the AUROC of rSIG was 0.635 (95% CI, 0.552–0.719; *p*-value, < 0.001). Even TRISS, which had the best prediction accuracy, showed similar results in healthy and cardiovascular disease populations (AUROC in healthy vs. cardiovascular disease group: 0.887 vs. 0.705). Medication is an important confounding factor that impairs the prediction accuracy of these scoring systems.

Motor vehicle collision-related injury commonly caused multitrauma injury. Multitrauma injury involving both hemorrhagic shock and traumatic brain injury is common, particularly in Motor vehicle collision-related injury, and may impair the prediction performance of rSIG. In our study, 413 patients (21.3%) were multitrauma injury patients, and had a higher proportion of severe TBI (19.4 vs 11.2%), more severe injury [ISS ≥ 16, (49.4% vs 38.0%)], longer total LOS (median of LOS: 11 vs 8), and higher proportion of ICU admission (76.5% vs 66.9%) than isolated TBI group (Supplementary Table 1). The multitrauma injury patients also have higher SI, MSI, ISS, and NISS score; but lower rSIG, RTS, and TRISS score. There was no significant difference of mortality between isolated TBI and multitrauma injury groups. In the motor vehicle collision-related injury group, the prediction performance of rSIG was better than SI, MSI and ASI with AUROC of 0.806 under 95% CI: 0.737–0.876. Sensitivity analysis of the multitrauma injury group showed rSIG had better prediction accuracy of mortality in the mixed TBI group than SI, MSI, and ASI with AUROC of 0.792 (95% CI: 0.700–0.883). Falls are another common injury mechanism and, in addition to traumatic brain injury, can cause of traumatic spinal cord injury (tSCI). tSCI affects hemodynamic stability from spinal shock, which is characterized by hypotension in the setting of a normal heart rate. Up to 20% of cervical level injuries patients have neurogenic shock, while bradycardia occurs in nearly all patients with severe spinal cord injuries (25, 26). Although we did not have the record of definite diagnosis of spinal cord injury in our database, the subgroup analysis of falls was conducted to investigate predictive score performance in TBI with risk of spinal cord injury. In this group, all prediction scoring systems showed reduced AUROC compared to the total TBI group, but the prediction performance of rSIG remained better than SI, MSI, and ASI with AUROC of 0.652 (95% CI: 0.586–0.717). Based on these results, rSIG is a rapid and accurate tool for field triage and prediction of mortality in the multitrauma injury group and patients with mechanisms of motor vehicle collision-related injury and falls. However, more robust studies to verify the results of our study are still required.

This study had several strengths. First, our study compared four major shock index scoring systems for mortality prediction in a population with TBI, and assessed different subgroups. Second, we focused on an exclusive Asian population, which has not been widely analyzed in previous studies. Racial/ethnic variation can change the normal range of blood pressure (27, 28), and the cohort studied here may be more reflective of the large global Asian population.

However, this study had some limitations. First, the time of rSIG measurement from injury to hospital admission is an important confounding factor, particularly in cases of delayed admission or prolonged prehospital times. Chi-Hsin Chen et al. using (29) Pan-Asia Trauma Outcomes Study (PATOS) database showed prehospital time was positively associated with poor functional outcomes in injured patients. In delayed admission or prolonged prehospital times groups, the different rSIG measurement time may lead to different prediction results. In our study, there was lack the detail time of rSIG measurement, including interhospital transfers, delayed admission or prolonged prehospital times. Although the transport interval and prehospital interval in Taipei was shorter than other countries (Median duration: 7 and 23 min), variable rSIG measurement times in injury patients may decrease prediction accuracy (30). Second, vital signs were obtained only once when the patient arrived at the ED. The fluctuating nature of vital sign after injury makes accurate vital signs assessment difficult. This is particularly true of GCS, which is a key component of the rSIG score. Furthermore, accurate GCS measurement is difficult in certain patient populations, such as those with intellectual disabilities, or under the influence of alcohol or drugs. Therefore, a single data point of vital signs and GCS in a progressive injury may not always reflect the clinical condition. Many studies have been suggested using a series of follow-up scoring systems to improve prediction accuracy, such as delta SI (31, 32). Series rSIG measurement not only reflects the disease progression but also decreases the impairment of measure time variation. The trend of rSIG would be more clinically relevant. Third, the difficulty of assessing GCS in certain patient populations, such as those with intellectual disabilities, alcohol or drug use, may limit the application of rSIG score. In future, more objective clinical features, such as injury mechanism and neurological sign, may be considered to include in prediction model to increase prediction performance. Forth, our study did not measure the disability scores, such as Modified Rankin Scale, EQ-5D-5L, or Instrumental activities of daily living, for mobility after discharge or during follow up period. In response to peer review, we provided the discharge deposition status in our study as post TBI functional outcome (Supplementary Table 2).

Another essential limitation is that some clinical data was missing owing to the retrospective nature of this study. In our study, cases were not included in calculation of national estimates of a value when that value was missing. The numbers of cases with missing values for diastolic blood pressure (1 cases), respiratory rate (7 cases), MSI (1 cases), RTS (7 cases, due to missing value of RR), TRISS (7 cases, due to missing value of RR) were trivial (totally 8 cases, 0.45%), so imputation was not considered necessary. And, we also think that it would be inappropriate to impute data of vital sign records, whereas it would be of less value to impute physiological variables other than vital sign. In addition, after reviewing the product information of blood pressure monitors, we defined the outlier of systolic blood pressure <60 or ≥280 mmHg, diastolic blood pressure <40 or ≥200 mmHg, respiratory rate <10 or ≥40 per minutes, and heart rate <40 or ≥200 mmHg. A sensitivity analysis was conducted after removed all the outliers. The results of sensitivity analysis did not change the final conclusion (Supplementary Tables 3, 4).

Finally, the event rate is an important factor impairing the ROC curve. In our study, we included 1,791 brain injury patients with only 9.3% positive case, which may cause ROC curves having an unstable shape and resulting in considerable risks of a type I error due to small sample size, especially in multiple comparisons. Also, this is a retrospective analysis of a rather homogenous population from Taiwan. The retrospective nature of the study is a limitation, as it was not conducted in real-time and was limited by study population. The application of our results on mixed cohorts or in other settings needs further consideration. In future, large sample size studies are necessary to validate our results.

## Conclusion

We found that the TRISS was still the best predictor of all scoring systems in TBI patients, including ISS, RTS, NISS, SI, MSI, ASI, and rSIG, in all subgroup populations. Compared to SI, MSI, and ASI, rSIG had a better prediction of mortality than in the TBI population, especially in the moderate/severe-TBI population but not in the mild-TBI population. In the subgroup analysis, the prediction accuracy of mortality of rSIG was better in TBI with major trauma (ISS ≥ 16), motor vehicle collisions, fall injury, and healthy and cardiovascular disease populations. The rSIG also has a higher prediction effect, as compared to SI, MSI, and ASI, both in the older population and the younger population. Overall, TRISS had the best predictive accuracy than all scoring system, but rSIG is easier to calculate and may be effective for prediction of mortality in emergency departments and during prehospital care.

## Data availability statement

The original contributions presented in the study are included in the article/Supplementary material, further inquiries can be directed to the corresponding author.

## Ethics statement

The studies involving human participants were reviewed and approved by Taipei Tzu Chi Hospital. The ethics committee waived the requirement of written informed consent for participation.

## Author contributions

P-CL, C-YL, and M-YW contributed to conception and design of the study. Y-TH, Y-LC, and C-YC organized the database. I-ST and T-HH performed the statistical analysis. M-YW wrote the first draft of the manuscript. Y-LC, D-SC, and G-TY wrote sections of the manuscript. All authors contributed to manuscript revision and read and approved the submitted version.
