# Supplementary material for: Shock index, modified shock index, age shock index score, and reverse shock index multiplied by Glasgow Coma Scale predicting clinical outcomes in traumatic brain injury: Evidence from a 10-year analysis in a single center
Source: Front Med (Lausanne). 2022 Nov 22;9:999481. doi: 10.3389/fmed.2022.999481 (PMC9723330; doi:10.3389/fmed.2022.999481)
Supplement: Supplementary file 1 [file Data_Sheet_1.docx]

Article

Shock index, modified shock index, age shock index score, and reverse shock index multiplied by glasgow coma scale predicting clinical outcome in traumatic brain injury: evidence from a 10-year analysis in a single-center

**Supplement**

**Supplement Table 1.** Demographics of the mixed and isolated traumatic brain injury population.

**Supplement Table 2.** Discharge deposition status of traumatic brain injury.

**Supplement Table 3.** Clinical outcome prediction by univariable logistic regression after exclusion of outliers.

**Supplement Table 4.** Clinical outcome prediction by multivariable logistic regression after exclusion of outliers.

Supplement Table 1. Demographics of the mixed and isolated traumatic brain injury population.

| Characteristics | Total patients | Mixed TBI | Isolated TBI | p-value |
| --- | --- | --- | --- | --- |
| Patient number, n (%) | 1791(100.0%) | 413(23.1%) | 1378(76.9%) |  |
| Age (years), mean±SD | 63.33±19.82 | 56.90±20.98 | 65.26±19.05 | <0.001 |
| Gender, n (%) |  |  |  | 0.169 |
| Male, n (%) | 1083(60.5%) | 262(63.4%) | 821(59.6%) |  |
| Female, n (%) | 708(39.5%) | 151(36.6%) | 557(40.4%) |  |
| GCS, median (IQR) | 15(13-15) | 15(11-15) | 15(14-15) | <0.001 |
| Traumatic brain injury (TBI) |  |  |  | <0.001 |
| Mild TBI, n (%) | 1375(76.8%) | 283(68.5%) | 1092(79.2%) |  |
| Moderate TBI, n (%) | 182(10.2%) | 50(12.1%) | 132(9.6%) |  |
| Severe TBI, n (%) | 234(3.1%) | 80(19.4%) | 154(11.2%) |  |
| Scoring systems |  |  |  |  |
| Shock index, mean±SD | 0.59±0.21 | 0.67±0.28 | 0.57±0.18 | <0.001 |
| MSI, mean±SD | 0.82±0.27 | 0.92±0.36 | 0.79±0.23 | <0.001 |
| Age SI, mean±SD | 36.15±14.85 | 36.03±16.82 | 36.18±14.21 | 0.870 |
| rSIG, mean±SD | 24.72±10.08 | 21.76±10.71 | 25.61±9.71 | <0.001 |
| ISS, median (IQR) | 11(9-16) | 14(11-22) | 9(9-16) | <0.001 |
| ISS ≥ 16, n(%) | 728(40.6%) | 204(49.4%) | 524(38.0%) | <0.001 |
| RTS, mean±SD | 7.30±1.24 | 7.03±1.51 | 7.40±1.08 | <0.001 |
| NISS, mean±SD | 15.73±9.63 | 20.16±11.72 | 14.40±8.47 | <0.001 |
| TRISS, mean±SD | 0.90±0.20 | 0.85±0.25 | 0.91±0.17 | <0.001 |
| Clinical outcome |  |  |  |  |
| LOS days, median (IQR) | 9(5-19) | 11(6-23) | 8(5-18) | <0.001 |
| ICU Admission, n(%) | 1238(69.1%) | 316(76.5%) | 922(66.9%) | <0.001 |
| ICU Readmission, n(%) | 33(1.8%) | 12(2.9%) | 21(1.5%) | 0.092 |
| ICU days, median (IQR) | 3(0-6) | 3(1-7.5) | 3(0-5) | <0.001 |
| ICU LOS ≥14 days, n(%) | 79(4.4%) | 23(5.6%) | 56(4.1%) | 0.191 |
| Operation, n(%) | 443(24.7%) | 151(36.6%) | 292(21.2%) | <0.001 |
| Reoperation, n(%) | 72(4.0%) | 34(8.2%) | 38(2.8%) | <0.001 |
| Death, n(%) | 72(4.0%) | 42(10.2%) | 124(9.0%) | 0.498 |

Abbreviations: GCS, Glasgow Coma Scale; MSI, modified shock index; ASI, age-adjusted shock index; rSIG, reverse shock index multiplied by Glasgow Coma Scale; ISS, injury severity score; RTS, revised trauma score; NISS, New injury severity score; TRISS, Trauma and Injury Severity Score; LOS, length of stay; ICU, intensive care unit.

Supplement Table 2. Discharge deposition status of traumatic brain injury.

| Characteristics | Mild TBI  (GCS ≥13) | Moderate TBI  (13>GCS ≥9) | Severe TBI  (9>GCS) | p-value |
| --- | --- | --- | --- | --- |
| Patient number, n (%) | 1375(76.8%) | 182(10.2%) | 234(13.1%) |  |
| Survival discharge status^‡^, n(%) |  |  |  | <0.001 |
| Recovery | 1096(79.7%) | 99(54.4%) | 78(33.3%) |  |
| Rehabilitation | 118(8.6%) | 35(19.2%) | 41(17.5%) |  |
| Transferred | 53(3.9%) | 11(6.0%) | 14(6.0%) |  |
| Death, n(%) | 56(4.1%) | 25(13.7%) | 85(36.3%) |  |

^‡^ There were 80 patients missing the discharge status.

Supplement Table 3. Clinical outcome prediction by univariable logistic regression after exclusion of outliers.

| Characteristics | Odds ratio of death | | |  | Odds ratio of ICU admission | | |  | Odds ratio of prolonged stay | | |
| --- | --- | --- | --- | --- | --- | --- | --- | --- | --- | --- | --- |
|  | OR | 95% CI | p–Value |  | OR | 95% CI | p–Value |  | OR | 95% CI | p–Value |
| Vital sign |  |  |  |  |  |  |  |  |  |  |  |
| SBP | 1.007 | 1.003-1.012 | 0.002 |  | 1.004 | 1.001-1.007 | 0.013 |  | 1.004 | 0.997-1.010 | 0.287 |
| DBP | 0.999 | 0.990-1.008 | 0.853 |  | 1.002 | 0.996-1.008 | 0.530 |  | 0.985 | 0.972-0.998 | 0.024 |
| RR | 0.961 | 0.891-1.037 | 0.303 |  | 1.039 | 0.992-1.087 | 0.106 |  | 1.079 | 0.985-1.183 | 0.101 |
| HR | 1.017 | 1.009-1.025 | <0.001 |  | 1.011 | 1.005-1.017 | <0.001 |  | 1.012 | 1.001-1.024 | 0.036 |
| Scoring systems |  |  |  |  |  |  |  |  |  |  |  |
| Shock index | 1.813 | 0.894-3.679 | 0.099 |  | 1.262 | 0.766-2.079 | 0.360 |  | 1.812 | 0.683-4.806 | 0.232 |
| MSI | 2.027 | 1.154-3.563 | 0.014 |  | 1.452 | 0.958-2.201 | 0.079 |  | 2.370 | 1.125-4.989 | 0.023 |
| ASI | 1.018 | 1.009-1.028 | <0.001 |  | 1.006 | 0.999-1.013 | 0.113 |  | 1.010 | 0.996-1.024 | 0.151 |
| rSIG | 0.912 | 0.896-0.929 | <0.001 |  | 0.967 | 0.957-0.977 | <0.001 |  | 0.955 | 0.933-0.977 | <0.001 |

SBP, systolic blood pressure; DBP, diastolic blood pressure; RR, respiration rate; HR, heart rate; GCS, Glasgow Coma Scale; MCV, motor vehicle collision; MSI, modified shock index; ASI, age-adjusted shock index; rSIG, reverse shock index multiplied by Glasgow Coma Scale.

| Characteristics | Adjusted odds ratio of death | | |  | Adjusted odds ratio of ICU admission | | |  | Adjusted odds ratio of prolonged stay | | |
| --- | --- | --- | --- | --- | --- | --- | --- | --- | --- | --- | --- |
|  | OR | 95% CI | p–Value |  | OR | 95% CI | p–Value |  | OR | 95% CI | p–Value |
| Scoring systems |  |  |  |  |  |  |  |  |  |  |  |
| SI | 1.483 | 0.707-3.112 | 0.297 |  | 0.935 | 0.525-1.665 | 0.820 |  | 1.491 | 0.551-4.029 | 0.431 |
| MSI | 1.584 | 0.863-2.905 | 0.138 |  | 1.157 | 0.727-1.841 | 0.540 |  | 1.961 | 0.901-4.270 | 0.090 |
| ASI | 1.008 | 0.997-1.020 | 0.147 |  | 1.004 | 0.995-1.012 | 0.412 |  | 0.999 | 0.983-1.015 | 0.891 |
| rSIG | 0.932 | 0.912-0.953 | <0.001 |  | 0.983 | 0.970-0.995 | 0.007 |  | 0.973 | 0.946-0.999 | 0.048 |

Supplement Table 4. Clinical outcome prediction by multivariable logistic regression after exclusion of outliers.

Co-variables used in the multivariable logistic regression included age, sex, activation of trauma team, injury mechanism, injury type, Glasgow Coma Scale, triage, and prehospital management. Every scoring system was put in the multivariable logistic regression separately from each other due to their strong collinearity. The age was not adjusted in ASI analysis, and GCS was also not adjusted in rSIG analysis due to strong collinearity.
